# Granulomatosis with Polyangiitis Presenting with Coronary Artery and Pericardial Involvement

**DOI:** 10.1155/2015/516437

**Published:** 2015-12-22

**Authors:** Rohit Dewan, Humberto E. Trejo Bittar, Joan Lacomis, Iclal Ocak

**Affiliations:** ^1^University of Pittsburgh Medical Center, Radiology Suite 200 East Wing, 200 Lothrop Street, Pittsburgh, PA 15213, USA; ^2^University of Pittsburgh Medical Center, Department of Pathology A711 Scaife Hall, 3550 Terrace Street, Pittsburgh, PA 15261, USA

## Abstract

Granulomatosis with polyangiitis is a systemic disease resulting in necrotizing vasculitis of small- and medium-sized vessels. Cardiac involvement is rare and when present usually manifests with pericarditis and coronary artery vasculitis. We report here a case of granulomatosis with polyangiitis involving the native coronary arteries, bypass graft, and pericardium with interesting imaging findings on contrast-enhanced CT and MRI. A 57-year-old man with a history of chronic headaches presented to the emergency room with syncope. Contrast-enhanced CT demonstrated extensive soft tissue attenuation around the native coronary arteries and bypass graft. Contrast-enhanced MRI demonstrated enhancing nodular soft tissue surrounding the coronary arteries, bypass graft, and pericardium. Pericardial biopsy revealed a necrotizing granulomatous pericarditis with vasculitis concerning for granulomatosis with polyangiitis. The patient demonstrated MPO-positive and PR-3 negative serologies. After being discharged on rituximab and prednisone, follow-up CT 3 years later showed significant improvement of the soft tissue thickening surrounding the coronary arteries, bypass graft, and pericardium.

## 1. Introduction

Granulomatosis with polyangiitis (GPA) is a form of systemic vasculitis with necrotizing granulomatous inflammation of the upper and lower respiratory tracts and kidneys [1]. GPA involves mainly small- and medium-sized vessels and can rarely manifest with cardiac involvement [2]. In these cases patients may present with pericarditis, myocarditis, valvular lesions, coronary arteritis, and conduction system defects with a prevalence ranging from 5 to 90% [3].

The literature concerning cardiac involvement is limited. The few case reports and general reviews show that the two most common histologic cardiac manifestations are pericarditis and coronary arteritis. The most frequent clinical manifestation is cardiac arrhythmias, typically supraventricular tachyarrhythmias. We report an unusual case of GPA initially presenting with cardiac involvement affecting the coronary arteries, bypass graft, and pericardium.

## 2. Case Report

A 57-year-old white male with history of Graves' disease with ophthalmopathy and coronary artery disease status after 3-vessel CABG presented to the ER after a syncopal episode during a doctor's office appointment. He had been having severe frontal headaches for several months with workup prior to this admission revealing an elevated ESR and bilateral temporal artery biopsies which were negative for giant cell arteritis. A lumbar puncture revealed elevated protein, increased opening pressure, elevated WBC, IgG index, and oligoclonal bands. A bone marrow biopsy performed for microcytic anemia was unremarkable.

**Figure 1:**
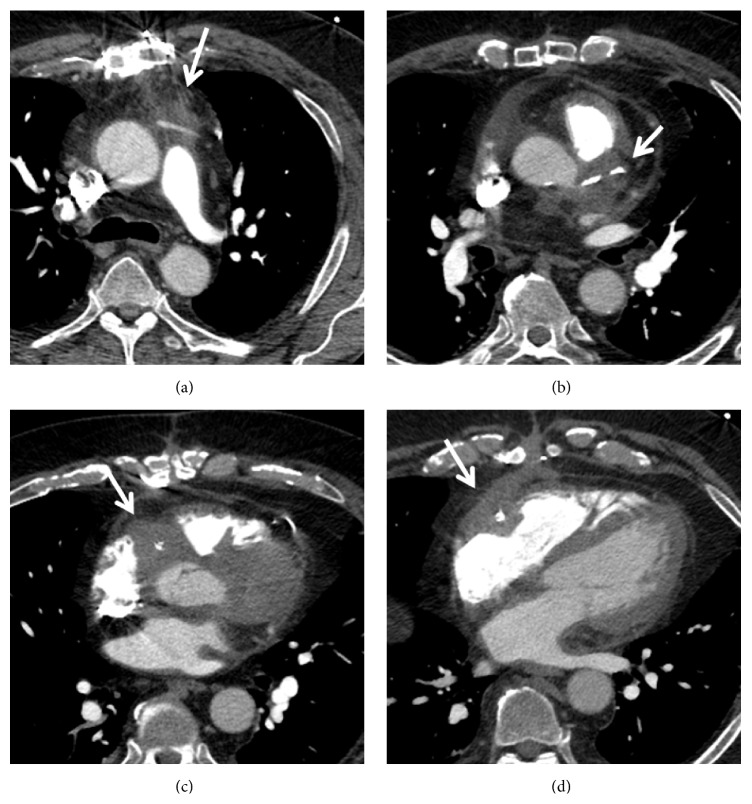
Contrast-enhanced computed tomography shows subsequent images through heart in the patient with granulomatosis with polyangiitis, extensive soft tissue attenuation around the coronary artery graft (a), left main coronary artery (b), and right coronary artery (c, d).

**Figure 2:**
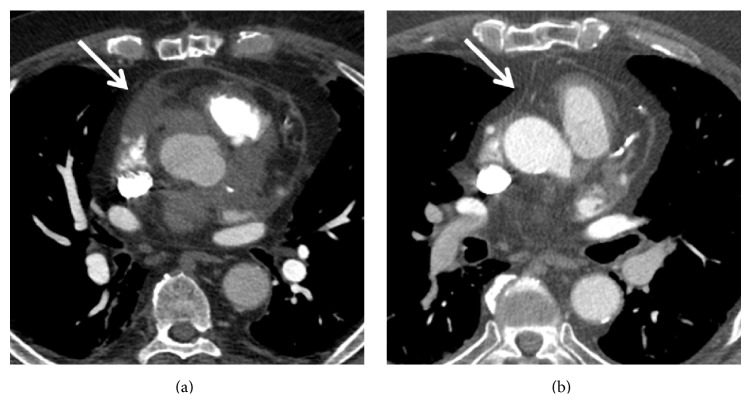
Contrast-enhanced computed tomography, subsequent images through heart in the patient with GPA show soft tissue attenuation around the pericardium (a). Resolution of pericardial thickening after the immunotherapy (b).

**Figure 3:**
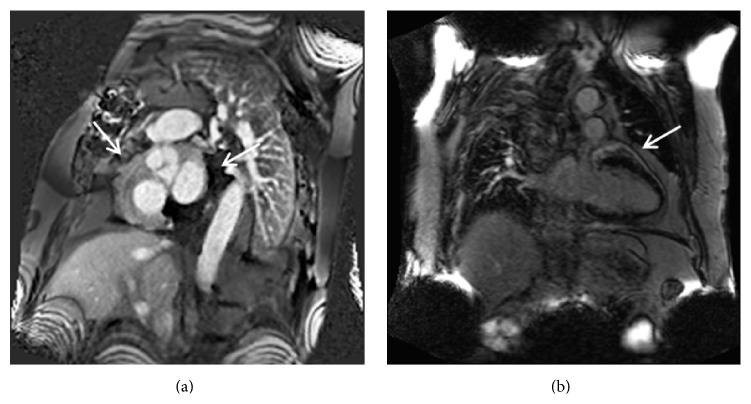
Cardiac granulomatosis with polyangiitis in a 57-year-old man. Phase sensitive inversion recovery images in the short axis (a) and long axis projections (b), 1 minute after the intravenous administration of contrast material show enhancing soft tissue thickening around coronary arteries (a) and pericardium (b).

**Figure 4:**
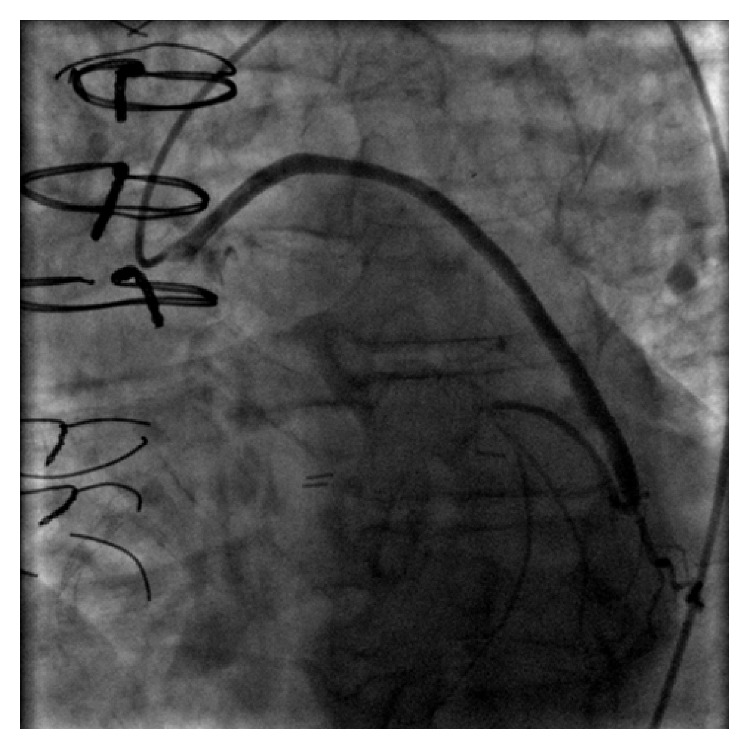
Conventional angiography shows a widely patent saphenous vein to obtuse marginal graft.

Given his history of syncope, a CT angiogram of the chest was performed to exclude a pulmonary embolism. No evidence of pulmonary embolism was seen; however, soft tissue attenuation around the coronary arteries, bypass graft, and pericardium raised concern for vasculitis (Figures 1 and 2). Further workup with cardiac MRI demonstrated enhancing soft tissue around the graft and coronary arteries with a nodular appearance of the pericardium (Figure 3). Conventional coronary angiogram revealed complete occlusion of LAD and right coronary artery although the bypass grafts were patent and there was no evidence of vasculitis (Figure 4).

**Figure 5:**
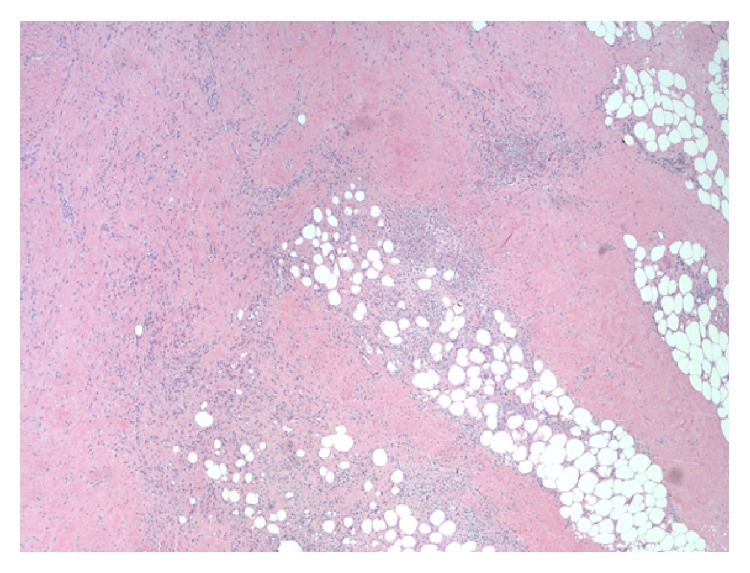
Pericardium with prominent scar tissue and nodular aggregate of monocytes and macrophages. Magnification 40x.

**Figure 6:**
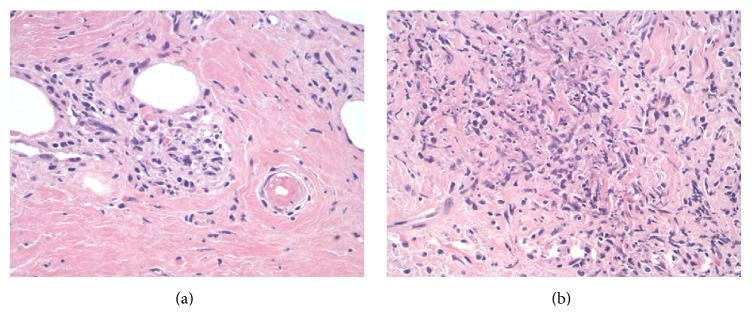
(a) Higher magnification of nodular areas. Leukocytoclasis is evident by the presence of vascular damage caused by nuclear debris from infiltrating neutrophils. Magnification 200x. (b) An earlier example of leukocytoclasis where the contour of the small vessel is still evident (arrow). Magnification 400x.

A pericardial biopsy demonstrated dense scar tissue associated with a mononuclear infiltrate comprised mostly of nodular aggregates of monocytes and macrophages (Figure 5). Within the nodular aggregates, myeloperoxidase-positive neutrophilic infiltration was localized to very small venules and capillaries with associated leukocytoclasis (Figure 6(a)). This granulomatous capillaritis with leukocytoclasis and peculiar mononuclear infiltrate was suggestive of GPA (Figure 6(b)). Although not as sensitive and specific as c-ANCA, positive p-ANCA serologies as in our patient have been associated with GPA.

**Figure 7:**
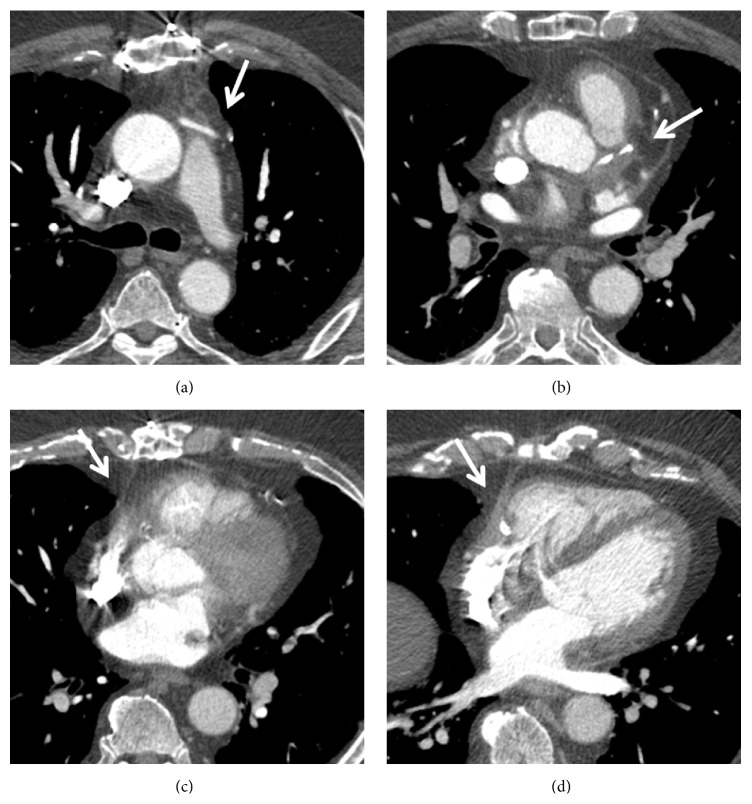
Contrast-enhanced computed tomography with subsequent images through the heart, in patient with granulomatosis with polyangiitis, shows resolution of soft tissue attenuation around the coronary artery graft (a), left main coronary artery (b), and right coronary artery (c, d) after the immunosuppressive therapy.

Myeloperoxidase associated ANCA vasculitis with orbital pseudotumor and pericardial involvement was considered to be the most likely diagnosis. He was discharged after being placed on prednisone and rituximab. Subsequent contrast-enhanced chest CT 3 years later showed significant improvement of the soft tissue thickening around the coronary arteries, bypass graft, and pericardium (Figure 7).

## 3. Discussion

GPA affects multiple organ systems and may involve any part of the body. The upper respiratory tract is involved in nearly all patients. In addition, a vast majority of patients with GPA will also have pulmonary (90%) and renal (80%) involvement [2]. Elevation of serum cytoplasmic ANCA (c-ANCA) titers, usually directed toward proteinase-3 and myeloperoxidase (found in neutrophils), occurs in up to 90% of patients with active GPA [2]. Common thoracic radiologic findings include pulmonary nodules, masses, ground-glass opacities, and consolidation. Airway, mediastinal, cardiac, and pleural involvement are less common. CT is the imaging modality of choice for diagnosis, surveillance, and follow-up in patients with GPA [2].

Cardiac involvement is relatively rare in GPA even though autopsy results show that GPA related cardiac abnormalities are present in one-third of patients [4]. Cardiac involvement in GPA is secondary to necrotizing vasculitis with granulomatous infiltrates. Pericarditis and coronary vasculitis are the most frequent findings (50% of cases), but myocarditis, endocarditis, and conduction system abnormalities have also been described [5]. In a study by Hoffman et al., cardiac involvement—predominantly pericarditis—was seen in 10 (6%) of 158 patients with GPA [5]. Coronary involvement is rare and is characterized by coronary arteritis and subsequent coronary artery thromboembolism [6]. Myocardial ischemia can result from vasculitic occlusion of small- and medium-sized coronary arteries [2].

The coronary arteries are frequently involved in systemic arteritis. The inflammatory infiltrate damages the intima and may trigger the occurrence of coronary thrombosis. Morbini et al. report an extreme example of an elderly female with intimal inflammation in multiple sites of a coronary tree with and without atherosclerosis which triggered coronary thrombosis. She died after cardiac arrest from a clinically unrecognized systemic autoimmune-inflammatory disorder with necrotizing arteritis. Autopsy showed findings typical of GPA and systemic arteritis with fibrinoid necrosis and systemic arteritis. Although there were no clinical signs of cardiac involvement, the coronary arteries showed inflammation associated with multiple mural and occlusive thrombi [7].

Ohkawa et al. reported a case of generalized GPA with extensive cardiac involvement at autopsy. Necrotizing angiitis and severe granulomatous inflammatory foci affected the common bundle of His and right bundle branch in addition to the myocardium [8].

Cardiac valvular involvement is an uncommon manifestation of GPA. Espitia et al. reported the case of a 60-year-old woman with severe inflammatory aortic and mitral valvular involvement characterized by GPA with typical histopathological valvular lesions. Cardiac valvular involvement is a rare and potentially fatal complication of GPA and may misleadingly suggest infectious endocarditis [9].

Currently, multiple imaging modalities are increasingly used as first-line noninvasive diagnostic tools to assess target-organ involvement in GPA, although biopsy with pathological testing represents the gold standard [10]. In our case CTA was performed to exclude pulmonary embolism and showed extensive soft tissue thickening around the coronary arteries, bypass graft, and pericardium. Pathology confirmed GPA. The lungs were normal.

To the best of our knowledge this is the first case report showing involvement of a bypass graft, native coronary arteries, and pericardium as the initial manifestation of GPA.

## 4. Conclusion

In conclusion, cardiac involvement of GPA is rare. It must be included in the differential diagnosis when soft tissue thickening involves the coronary arteries and pericardium, even when no pulmonary or airway findings are identified.

## References

[1] Jennette J. C., Falk R. J., Bacon P. A. (2013). 2012 Revised International Chapel Hill consensus conference nomenclature of vasculitides. *Arthritis & Rheumatism*.

[2] Martinez F., Chung J. H., Digumarthy S. R. (2012). Common and uncommon manifestations of wegener granulomatosis at chest ct: radiologic-pathologic correlation. *Radiographics*.

[3] McGeoch L., Carette S., Cuthbertson D. (2015). Cardiac involvement in granulomatosis with polyangiitis. *The Journal of Rheumatology*.

[4] Yi E. S., Colby T. V. (2001). Wegener's granulomatosis. *Seminars in Diagnostic Pathology*.

[5] Hoffman G. S., Kerr G. S., Leavitt R. Y. (1992). Wegener granulomatosis: an analysis of 158 patients. *Annals of Internal Medicine*.

[6] Parry S. D., Clark D. M., Campbell J. (2000). Coronary arteritis in wegener's granulomatosis causing fatal myocardial infarction. *Hospital Medicine*.

[7] Morbini P., Dal Bello B., Arbustini E. (1998). Coronary artery inflammation and thrombosis in Wegener's granulomatosis-polyarteritis nodosa overlap syndrome. *Giornale Italiano di Cardiologia*.

[8] Ohkawa S.-I., Miyao M., Chida K. (1999). Extensive involvement of the myocardium and the cardiac conduction system in a case of Wegener's granulomatosis. *Japanese Heart Journal*.

[9] Espitia O., Droy L., Pattier S. (2014). A case of aortic and mitral valve involvement in granulomatosis with polyangiitis. *Cardiovascular Pathology*.

[10] Florian A., Slavich M., Blockmans D., Dymarkowski S., Bogaert J. (2011). Cardiac involvement in granulomatosis with polyangiitis (wegener granulomatosis). *Circulation*.

